# HIV-1 Reservoir Association with Immune Activation

**DOI:** 10.1016/j.ebiom.2015.08.025

**Published:** 2015-08-16

**Authors:** Alejandro Vallejo

**Affiliations:** Laboratory of Immunovirology, Department of Infectious Diseases, Institute Ramón y Cajal of Health Research (IRyCIS), Universitary Hospital Ramón y Cajal, Madrid, Spain

**Keywords:** HIV-1 DNA load

## Abstract

In this issue of *EBioMedicine*, Ruggiero and colleagues describe immune activation biomarkers associated with the size of the HIV reservoir in a carefully designed cross-sectional study. The cohort consists of a homogeneous sample of HIV-1-infected patients with long-term plasma HIV-1 RNA suppression under antiretroviral treatment (ART). It is crucial to explore the potential utility of biomarkers that are easier (less labor intensive, less expensive) to measure than integrated HIV DNA load, in order to quickly and accurately quantify cellular reservoirs of HIV.

## 1 Commentary

While current combination antiretroviral treatment (ART) successfully suppresses human immunodeficiency virus (HIV) replication, it cannot eradicate the virus since HIV efficiently resides latently in some cellular reservoirs, mainly resting CD4 T cells but also dendritic cells and other cell types. It is known that HIV establishes a reservoir of replication-competent integrated provirus early after infection that is unresponsive to ART (Chavez et al., 2015; Martínez-Bonet et al., 2015; Siliciano and Siliciano, 2015). Although the mechanism of HIV persistence is not fully understood, proposed models focus on the continuous replenishment of the reservoir through ongoing virus production, perhaps in sites where ART penetration or activity is suboptimal. As an alternative, maintenance of the reservoir during ART can also occur through proliferation of latently infected cells. Models have also been proposed to explain persistent immune activation despite ART, and include HIV-induced gut damage that allows translocation of microbial products into the systemic circulation. Other persistent infections, such as cytomegalovirus (CMV) or Epstein–Barr virus (EBV), have also been linked to persistent immune activation. The measurement of HIV reservoir includes the accurate quantification of integrated HIV-1 DNA in peripheral blood and other cells or tissues (Archin et al., 2014).

The study by Ruggiero and colleagues describes factors associated with the size of the HIV reservoir in a very well designed cross-sectional study of a carefully selected and homogeneous sample of HIV-1-infected patients with long-term plasma HIV-1 RNA suppression under ART (Ruggiero et al., 2015). It is important to note that the study cohort was uniform with respect to the initial first-line antiretroviral regimen and absence of viral blips for up to 14 years. The size of the reservoir was measured by the quantification of integrated HIV-1 DNA as well as by 2-long terminal repeat circular HIV-1 DNA. Investigated factors associated with the reservoir included residual plasma HIV-1 RNA and markers of immune activation on CD4 and CD8 T cells, and also the quantification of soluble CD14 as a marker of monocyte/macrophage activation. The main finding of this study was a positive association between the levels of integrated HIV-1 DNA in peripheral blood and the frequency of CD8 T cells expressing HLA-DR/DP/DQ. Furthermore, the lack of association between integrated HIV-1 DNA load and CD8 and CD4 cells expressing CD38 indicates that the association involves a distinct T cell activation profile. The immune activation marker, HLA-DR/DP/DQ, can therefore indirectly estimate the HIV reservoir in eradication strategies. This paper contributes to evidence that a complex interplay between HIV-1 persistence and immune activation continues over many years despite stably suppressive ART.

Although the function of CD8 T cells expressing HLA-DR/DP/DQ (without CD38) needs to be further studied, both regulatory and effector functions may play a role. Crucially, CD8^+^HLADR/DP/DQ^+^ cells may stimulate HIV-infected CD4 cells, causing expansion of the reservoir. As the authors mentioned, it will be important to determine the antigenic specificity of these cells and any possible activity against persistent viruses such as CMV and EBV. These infections were not commonly detected in the cohort studied and this observation would suggest immunological control. As the authors recommend, it will be important to study CD8^+^CD38^−^HLA-DR/DP/DQ^+^ cells specifically, as they are preferentially generated in response to low antigenic stimulation and may play a role as elite controllers, suppressing HIV replication as well as clearing hepatitis C infection.

New approaches to eradicate HIV infection, or at least to achieve a “functional cure”, include the depletion of persistent infection, improvement of the immune response and the disruption of latent infection (Chun et al., 2015). For this purpose, it is crucial to develop accurate tools to directly quantify cellular reservoirs of HIV (Crooks et al., 2015), or exploit immunological biomarkers to determine HIV persistence by providing an indirect measure of the size of the reservoir as described by Ruggiero and colleagues. In both cases, validation in multiple independent cohorts is desirable in order to confirm their scientific utility.

## Disclosures

The author declares no conflicts of interest.
